# Profiling Speech and Language Therapy Delivery in Custodial Youth Justice Settings in England: A Case Study Series

**DOI:** 10.3390/healthcare14172882

**Published:** 2026-09-07

**Authors:** Kim Turner, Judy Clegg, Sarah Spencer

**Affiliations:** 1 Health Professions, Manchester Metropolitan University, Manchester M15 6GX, UK; 2 School of Allied Health Professions, Pharmacy, Nursing and Midwifery, University of Sheffield, Sheffield S10 2TN, UK

**Keywords:** communication, language, criminal justice, case series

## Abstract

Background: A high prevalence of speech, language and communication needs are reported amongst individuals in contact with the criminal justice system. Speech and language therapists are increasingly employed to support people within prisons. As this is a relatively new area of clinical practice, there is limited research evidence to inform service delivery. Case studies are a first step in providing evidence of the effectiveness of speech and language therapy within a custodial environment. This study aims to profile the delivery of speech and language therapy in custodial youth justice settings in England, identifying the facilitators and barriers affecting these services. Method: Speech and language therapists working within two custodial youth justice settings in England described examples of speech and language therapy intervention they delivered in their settings (four case studies from one setting and two case studies from another). Six case studies are presented, profiling speech and language therapy for six male participants, aged 17–21. A case study template included background information, referral information, assessment details, intervention details, and session descriptions. Results: A variety of intervention approaches were used based on the individual’s needs. These were all reported following a framework developed in an earlier study. The intervention duration ranged between 25 and 510 min, consisting of 1–13 sessions. Intervention was provided both in individual and group settings and employed functional and impairment-based approaches. The outcomes of intervention were: an increase in self-awareness of strengths and needs, use of self-advocacy strategies, learning and using strategies to support effective communication, and a self-reported increase in well-being and self-esteem. Discussion: This case series highlights the range of interventions and approaches currently used in custodial youth justice settings in England. Further research is needed to explore speech and language therapy’s role in contributing to functional outcomes and its impact on mental health, well-being, and recidivism rates.

## 1. Introduction

A high prevalence of communication difficulties is reported amongst individuals in contact with the criminal justice system (CJS) [1]. An estimate of 9.92% has been reported for communication difficulties [2] in the UK paediatric population, with 40–60% of these persisting across the life course [3]. Prevalence of communication difficulties for those involved in the criminal justice system has risen steeply with a figure of 60% oft quoted (ranging from 19% to over 80%) [4]. Research included in the Anderson et al. [4] review illustrates an increased prevalence across the full breadth of communication—including speech, language use and understanding, and social skills. Over 1.5 million people had contact with the criminal justice system in England and Wales in 2025 [5]. Using the prevalence figures above, this equates to 900,000 individuals with communication needs accessing the criminal justice system in one year across just England and Wales.

The criminal justice system places high demands on individuals’ communication skills at each step, from engaging with the police and attending court to engaging with sentence demands. In a custodial setting individuals must learn the routine of the new environment, interact with a multitude of new people, attend education or work settings, and complete rehabilitation programmes. All of these tasks require effective communication skills [6], with communication difficulties linked to adverse outcomes and delayed progress through the system [7].

Speech and language therapists are increasingly employed to support people within prisons, with between 10 and 20% of UK prisons having access to this provision [8]. As this is a new area of clinical practice, there is limited research evidence to inform service delivery. It is reported that, in custodial settings, speech and language therapists provided assessment, intervention and training both directly and indirectly [9]. There is some evidence supporting the positive impact of speech and language therapy (SLT) interventions with adolescents and young adults [10,11]. As a new field of practice, there is minimal evidence for the efficacy of SLT interventions with young people in contact with justice services, although clinical reports support the benefit [12]. A study conducted within community youth justice in England [13] did demonstrate improvements in the individuals’ communication skills and also engagement with other services [14]. Within the custodial population, there is currently no published evidence from the UK. There exists some grey literature detailing individual case studies supporting the potential benefits of SLT in this setting [15,16]. Research conducted in a custodial youth justice setting in Australia provided evidence for the potential to deliver effective SLT interventions [17]. This study demonstrated direct benefits for some of the individuals with language difficulties and further indirect benefits in staff communication [18]. The interventions were delivered within the context of a research study rather than an established speech and language therapy service, and this limitation is acknowledged within the paper [17]. The authors recommended that this should be explored. This case series aimed to explore how speech and language therapy interventions are delivered within established prison SLT services in England.

The study aims to profile speech and language therapy interventions in custodial youth settings in England. The study asks the following research questions.

(1) How are speech and language therapy services delivered in custodial youth settings in England?
(2) What are the key facilitators and barriers experienced by these services?

## 2. Method

### 2.1. Design

A case series design was used to collect real-world data on examples of speech and language therapy interventions or delivery provided within custodial youth justice settings—these are prisons for children and young adults. All prisons in England housing individuals under 18 (*n* = 4) and having an SLT service were invited to take part (*n* = 3, further information about each of these services can be found in Turner, Spencer & Clegg [9]. Snow et al. [19] suggest single-case studies to gather initial evidence about the effectiveness of SLT in the criminal justice arena. Single-case studies also provide the opportunity to describe how interventions and support are provided. The case studies were collected from the therapists working in these services to: represent the variety of provision, offer the opportunity to look at variation between service providers, and also pragmatically minimise the time required from each clinician and the potential impact on service delivery.

Ethical permission was granted for this project as part of a larger study from the Institutional Review Board (or Ethics Committee) of the NHS Health Research Authority (209118, 7 April 2017) as well as the University of Sheffield Research Management System (149882, 20 February 2017).

### 2.2. Participants

Speech and language therapists:

There are four prisons in England housing individuals under 18, three of these having an SLT service at the time of data collection. All three speech and language services initially agreed to participate, though one later withdrew due to the post being vacant. Six speech and language therapists from two custodial youth justice settings in England participated.

Young people within custodial youth justice settings:

Intervention with six males aged 17;10 to 21;05 is reported. Participants had a range of communication needs including, speech, language and communication, and they were seen for both assessment and therapy, either 1:1 or in a group setting. Further participant data is summarised in Table 1. All participants are referred to by a pseudonym to protect their anonymity.

## 3. Materials

The case study template (File S1) was based on Snow and Woodward [17]. The template included background information, referral information, assessment details, intervention details, and session descriptions (see Supplementary Material S1).

In an earlier phase of the wider study [20], information about service users was collected. A high proportion of individuals had special education needs (SEN, 46.7%), had been excluded from school (80+%), had had contact with mental health services (51.1%), had been identified as ‘looked after children’ (LAC, 42.2%), and came from a Black, Asian and Minority ethnic (BAME) background (71.1%). Previous research [21] also indicated an increased level of SLCN amongst individuals who had committed violent offences. Therefore, these fields were added to the case study template. An overview of the template is given in Table 2 (for the full template, see Supplementary Materials).

Any available feedback from the service users on their experience of SLT was also requested to give insight into the acceptability of the service provided.

### 3.1. Interventions

As this was an investigation of current practice, no limitations were set on the type of intervention offered, modality, dosage or frequency.

### 3.2. Data Analysis

As this series of case studies aimed to explore how services are delivered and any barriers to service delivery, no inferential statistics were employed; descriptive statistics are presented where appropriate. We present demographic and background information, intervention focus, engagement and outcome information, and informal participant and clinician reflections, where available. Pseudonyms are used throughout. The information provided by clinicians was reviewed, and language and structure were standardised to fit the reporting template.

## 4. Results

The following six case studies aim to provide more detail about how SLT is delivered in custodial youth justice settings in England and what types of approaches are used. Case studies 1–4 are from Service 2 and case studies 5 and 6 are from Service 3. Four case studies show a complete episode of care, while case study 4 has only one group session documented and case study 6 has incomplete information about the provision of therapy delivered by the Learning Support Assistant (LSA). The case studies cover a range of different communication difficulties and include assessment and intervention programmes. Case studies 1–5 report intervention completed with a speech and language therapist; case study 6 reports intervention delivered by a Learning Support Assistant (LSA) under the supervision of SLTs.

Participants have a higher incidence of additional needs and adverse factors than the general public (see Table 3), but the proportion of young people identified as LAC is lower than in the wider study group [20]. Background data for the case studies was collected by the respective clinicians from the health care records and from the client.

The average number of sessions offered was 8.3 (SD = 5.04) and the average number of sessions completed was 7.4 (SD = 4:03). There was an 87% (SD = 0.12) attendance rate overall. Intervention had a median duration of 345 min (range 25–630, SD = 208.5).

### 4.1. Freddie

Age: 18 years and 6 months.

Therapist: Fulltime SLT.

#### 4.1.1. Background Information

Freddie was a young black male whose mother died when he was young. He lived with various family members and was living at a hostel before coming into custody. Freddie had no formal qualifications and reported he did ‘bad’ at school. He left fulltime school around the age of nine and appears to have engaged in education intermittently since then. He reported he had a statement of Special Educational Needs and had 1:1 support in secondary education. Freddie had contact with mental health services and was reported to have ‘low normal intelligence, or even borderline, and likely to have developed antisocial personality traits.’ Freddie has a history of offending and a previous custodial sentence. His current offence is possession of an offensive weapon. He was awaiting transfer to an immigration centre.

#### 4.1.2. Referral

Referral source: Comprehensive Healthcare Assessment Tool [22] completed by an Assistant Psychologist.

Reason for referral: Stammering.

#### 4.1.3. Intervention Overview

An overview of intervention for Freddie is presented in Table 4, below.

#### 4.1.4. Assessment

##### Assessment Tools

Perceptions of Stuttering Inventory and Wright and Ayre Stuttering Self-Rating Profile (WASSP [23]).

##### Assessment Results

Perceptions of Stuttering Inventory: Freddie indicated he displays behaviours in all three of the assessed categories: ‘Avoidance’, ‘Expectancy’ and ‘Struggle’. He scored highest on ‘Struggle’ (17/20), suggesting much of his difficulty relates to saying words fluently. He scored 8/20 for ‘Avoidance’ and 12/20 for ‘Expectancy’.

WASSP: Freddie rated his stuttering behaviours as well as his thoughts and feelings about stammering. Freddie indicated that his feelings of embarrassment, anger, helplessness and frustration are ‘very severe’. He also indicated he has fairly strong negative thoughts before and after he stammers.

**Diagnosis:** Stammer.

#### 4.1.5. Therapy

##### Overview

Freddie presents with a stammer and was eager to seek support for this. Freddie reported he had previously worked with a SLT. However, Freddie could not recall any strategies taught or much of the work he completed. He recalled he was encouraged to slow down his rate of speech as well as practise breathing and relaxation techniques. Freddie engaged in seven therapy sessions to provide him with strategies to improve confidence and increase fluency; see Table 5. Freddie’s therapy was underpinned by acceptance and commitment therapy principles [24]. Freddie also presented with language difficulties. However, his primary concern was his stammering. Freddie required questions to be simplified and also benefitted from SLT checking his understanding.

#### 4.1.6. Outcomes

Freddie was motivated to engage in sessions and completed additional work between sessions. Freddie demonstrated that his views of stammering were changing through therapy; whereas at the outset he would seek to avoid and ignore others, he shared that his friend had stammered in a music video, and they left it in because they liked how it sounded. The therapy block was not completed as Freddie requested a break from therapy due to stress relating to immigration issues. Freddie was transferred to an immigration removal centre two weeks after this. This meant formal outcome measures were not completed.

### 4.2. Sam

Age: 21;05.

Therapist: Sam was first seen for assessment by another therapist before being seen by the volunteer SLT.

#### 4.2.1. Background Information

Sam was a softspoken Asian male; he had a calm demeanour, was polite and had good eye contact. He was highly motivated to attend regular speech, language and communication therapy sessions and was very aware of his interdental S. People would frequently ask him to repeat what he said. He liked rapping and therefore wanted his speech to sound as clear as possible. After a few weeks, Sam complained about his poor memory, linking this to having been a cannabis user. It was therefore agreed that memory assessment, exercises and strategies could be included in his therapy sessions. Further assessment showed mild memory impairment, of which Sam was very aware and keen to address. No information about family, education, mental health or previous offending was provided.

#### 4.2.2. Referral

Referral source: Unknown.

Reason for referral: Speech sounds.

#### 4.2.3. Intervention Overview

An overview of intervention for Sam is presented in Table 6, below.

#### 4.2.4. Assessment (Previous)

##### Assessment Tools

Phonological screening assessment [25] and The Clinical Evaluation of Language Fundamentals [26] Working Memory sub-tests were completed.

##### Assessment Results

The articulation screening test confirmed the only speech sounds needing intervention were the S and Z sounds in all phonetic contexts; both sounds were consistently interdental, with the tongue clearly visible. Working Memory was within normal limits (standard score = 91; percentile rank = 27); however, forward number recall was below average.

**Diagnosis:** Interdental S and Z.

#### 4.2.5. Therapy

##### Overview

Articulation therapy was built around producing the target sound in isolation and progressing upwards in a stepped approach (see Table 7), following the principle developed by Van Riper [27]. A mirror was used in early sessions to enable accurate production of the target sounds. The visual feedback enabled Sam to become accustomed to the kinaesthetic feedback of new tongue placements. Sam worked on new sound production between sessions with tasks being created jointly. Picture sequences and small objects were used for the memory work.

#### 4.2.6. Outcomes

Sam completed a course of speech, language and communication therapy, although he missed the final planned session. Sam’s mood fluctuated over the course of therapy; however, he maintained engagement. He developed good insights into his articulation and how to self-monitor and correct. His family noticed increased clarity, and he was generally more confident in expressing himself. Sam also learnt a range of strategies to help with memory tasks.

### 4.3. Brett

Age: 20;11.

Therapist: Lead SLT.

#### 4.3.1. Background Information

Brett was White British and an only child. He lived with his mother until the age of 16 and had not had any contact with his biological father. Brett reported he was close to his mother, but he felt she was not always there for him throughout his childhood, leading to him moving to supported accommodation at age 16. Brett attended a mainstream school for his primary education. At age 11, he transferred to a Pupil Referral Unit (PRU). Brett had a long history of contact with mental health services for a number of different issues including depression, anxiety, hearing voices, substance misuse and Emotionally Unstable Personality Disorder. There is also a record of contact with SLT services due to delayed language development. Brett has a history of offending. His current offence is robbery with physical violence against the person.

#### 4.3.2. Referral

Referral source: Primary care.

Reason for referral: Had started an autism assessment at the previous prison.

#### 4.3.3. Intervention Overview

An overview of intervention for Brett is presented in Table 8, below.

#### 4.3.4. Assessment

##### Assessment Tools

Autism Diagnostic Observation Scale (ADOS-2 [28]), developmental history and Adult Autism Quotient Questionnaire (AQ 50 [29]) were used.

##### Assessment Results

The Autism Diagnostic Observation Schedule (ADOS) was completed over two sessions. In this assessment, Brett did not meet the threshold for autism (10) or autism spectrum conditions (7), scoring four. During the assessment, Brett repeatedly spoke about his love of routines and how it upset him if his routine was broken. Brett also spoke about the difficulties he experienced talking to others; he said he found it hard to start conversations and he would interrupt a lot. Brett also discussed the fact he was not good with money and found it difficult to budget, which had led to him losing his housing in the past.

On the Adult Autism-Spectrum Quotient Questionnaire (AQ 50), Brett scored 37/50. A score over 32/50 is considered significant; 80% of individuals with ASC score 32+, whereas only 2% of controls do.

The results from the AQ assessments were indicative of autism. However, the results from the ADOS were not suggestive of a diagnosis of autism. During the assessment process, Brett disclosed he had experienced adverse childhood experiences (ACEs). It was felt his presentation was consistent with these experiences; therefore, a diagnosis of autism was not indicated.

**Diagnosis:** NA.

#### 4.3.5. Outcomes

Brett willingly engaged in the assessment process and appeared keen to receive a diagnosis. Brett appeared very anxious about his performance and whether he had gotten it ‘right’ even though it had been explained there were no right or wrong answers. Brett shared he found new people and new situations difficult. He was offered support for his childhood trauma, anxiety and social communication. Brett asked for the assessment report to be shared with his mother and prison officers on his unit. Although he declined intervention regarding his social communication skills, the assessment of his needs was an important component of his treatment, providing him and those around him with insight and understanding of how he interacts. He was willing to work with a psychology team on a formulation but not to discuss previous trauma.

### 4.4. Salim

Age: 21 years.

Therapist: Student SLT.

#### 4.4.1. Background Information

Salim is an orphaned refugee from Afghanistan. His first language is Dari (similar to Farsi). His spoken English is reported to have improved greatly in the past year, being at a functional level. He witnessed close family members being killed in an explosion by the Taliban. Salim suffered a traumatic journey to the UK via a people smuggler. Salim is reported to have started school in the UK at age 14. Salim resided on the inpatient unit to be in a safe space; he has an open Assessment, Care in Custody and Teamwork (ACCT). An ACCT is opened when an individual is deemed to present an immediate risk to themselves. Salim has experienced depression, Post-Traumatic Stress Disorder (PTSD) and suicidal thoughts since his trauma in Afghanistan. He spent a year in a secure mental health facility in 2015. He had ongoing psychology sessions whilst serving his sentence. He had never had a visitor in prison. There was no history of offending. His current offence was possession of an imitation firearm, with intent to cause violence.

#### 4.4.2. Referral

Referral source: From liaison with community psychiatric nurses on unit.

Reason for referral: Member of social communication group provided to all patients on the unit.

#### 4.4.3. Intervention Overview

An overview of intervention for Salim is presented in Table 9, below.

#### 4.4.4. Assessment

##### Assessment Tools

Communication questionnaire and discussion around goals were conducted. 

##### Assessment Results

He speaks English as an additional language. He sometimes struggles to use effective body language and eye contact. Salim requires additional time and encouragement to contribute in group settings and to express his needs. Salim identified goals he wished to achieve; these included working towards taking his driving theory test.

**Diagnosis:** Unspecified.

#### 4.4.5. Therapy

##### Overview

Salim attended the weekly on-unit social communication group. The theoretical underpinnings for this approach are unknown, see Table 10. He attended most of the weekly one-hour sessions whilst a resident on the unit.

#### 4.4.6. Outcomes

Salim had very good engagement and motivation in sessions. He made good progress within sessions and demonstrated good self-awareness. With support, he could identify his own skills in successful conversations, such as turn-taking and staying on topic. Salim could identify unfamiliar words in the driving test manual and attempt to explain their meaning. Salim learnt new vocabulary; learning was supported by gestures and contextual information. He was able to retain this vocabulary and define it in his own words.

### 4.5. Xavier

Age: 17 years 10 months.

Therapist: Part-time SLT.

#### 4.5.1. Background Information

Xavier came from a traveller background and moved regularly, leading to fragmented education. He attended education until the age of 15. Xavier did not have a SEN statement or EHCP. Xavier stated he had basic reading and writing skills. He had been attending education in custody and achieved entry level 2 qualifications in Maths and English. In the longer term, Xavier stated he wanted to be in employment and had aspirations of running his own handyman business. Xavier had engaged in bare-knuckle fist fighting and sustained a head injury. Xavier was reported to be a heavy cannabis user in the community. There was no information available regarding previous offending. His current offences are burglary with intent to steal, attempted burglary, robbery and theft from a shop.

#### 4.5.2. Referral

Referral source: CAMHS Occupational Therapist (OT) within the YOI.

Reason for referral: Concerns re: understanding.

#### 4.5.3. Intervention Overview 

An overview of intervention for Xavier is presented in Table 11, below.

#### 4.5.4. Assessment

##### Assessment Tools

Local communication screening tool (the screen consists of understanding time, days, months, and their sequencing; verbal reasoning; understanding word meanings; and listening and remembering information) was used. 

##### Assessment Results

Xavier was assessed as requiring 1:1 work to support him to tell the time using an analogue clock and support him when processing information. Information should be repeated and supported in a visual format to give him the best chance of understanding. It should be checked if he has understood what has been arranged, rather than assuming he has. He needed information to be broken down into chunks to help him process it. He also required prompts to get and maintain his attention. He could not tell the time on an analogue watch and reported he could not tell the time unless it was on the hour or half-past. He said a digital clock was easier for him.

**Diagnosis:** Unspecified.

#### 4.5.5. Therapy

##### Overview

The SLT handed information over to the education Learning Support Assistant (LSA) to deliver therapy to Xavier. It was recommended that therapy should focus on developing listening and attention skills, which would support his ability to understand and process verbal language. The theoretical underpinnings for this approach are unknown and there were no details on this therapy provided due to limited contact between the SLT and LSA.

#### 4.5.6. Outcomes

No information was provided.

### 4.6. Trent

Age: 18;01.

Therapist: Part-time SLT and LSA.

#### 4.6.1. Background Information

Trent was living in semi-independent living prior to coming into custody. He currently has no contact with family. There is a history of physical and sexual abuse and he was known to social services. Trent was permanently excluded from school in Year 7 (age 12) and did not obtain any formal qualifications. He did not have a SEN statement or EHCP. Trent was engaging in education in custody; this was 1:1 due to disruptive behaviour. Trent has ADHD for which he is being medicated. He reported substance use in the community and has a history of seizures. There was no information available regarding previous offending. His current offence is sexual assault.

#### 4.6.2. Referral

Referral source: CAMHS.

Reason for referral: Receptive language.

#### 4.6.3. Intervention Overview

An overview of intervention for Trent is presented in Table 12, below.

#### 4.6.4. Assessment

##### Assessment Tools

Local communication screening tool (the screen consists of understanding time, days, months, and their sequencing; verbal reasoning; understanding word meanings; and listening and remembering information) was used. 

##### Assessment Results

Primary areas of need were identified as vocabulary, memory and understanding.

**Diagnosis:** Unspecified.

#### 4.6.5. Therapy

##### Overview

The SLT handed information over to the education Learning Support Assistant (LSA) to deliver therapy to Trent, see Table 13. Again, the theoretical underpinnings for this approach are unknown. He was seen for four sessions in an educational setting by the LSA. He appeared reluctant to engage and, since starting a new course, had refused to engage; therefore, he was removed from the caseload.

#### 4.6.6. Outcomes

A number of inferential comprehension tasks were completed. At times Trent seemed to enjoy the challenge of answering questions based purely on the picture clues, but at other times, he really struggled with concentration and motivation and would flit between a variety of tasks. He showed a preference for ascertaining information from pictures rather than text.

The case studies demonstrate the range of work completed by SLTs in this setting. The case studies also serve to highlight the additional difficulties experienced by these individuals including ACEs, school exclusion and mental health difficulties. Clients were seen individually and in groups, for assessment and therapy, and for a range of speech, language and communication difficulties. The four case studies presented here, from a total of six with an intervention component, cover five areas: stammering, speech, social communication, memory and comprehension. The outcomes of the SLT input were functional and included an increase in self-awareness of strengths and needs, self-advocacy strategies, learning and using strategies to support effective communication, and an increase in well-being and self-esteem. There was a significant range in the type and quantity of input between case studies.

## 5. Discussion

The case studies detailed above show that speech and language therapy services provide both assessment and intervention through direct and indirect routes. Speech and language therapy services provide support for young people with a wide range of speech, language and communication needs. Young people and therapists report positive outcomes relating directly to speech, language and communication needs and more broadly to well-being. There appears to be a range of facilitators and barriers affecting these services. Where direct intervention was provided, there was clearer oversight of provision. Many of the young people had complex, traumatic backgrounds which would require careful consideration when planning the timing, environment, and target setting of service delivery. It is unclear from the information provided whether the quantity of input provided to each individual was based on service pressures, client preference, clinical need or other factors. Therapists working in criminal justice settings have been referred to as specialist-generalists. This appears a fitting title as they are required to work with a broad range of SLCN within a specialist field.

The four intervention case studies addressed five categories: stammering, speech, social communication, memory, and comprehension. As previously mentioned, there is one article [16] on the benefits of stammering therapy for this population; however, the study provided the intervention through telehealth, and therefore, this study is not directly comparable with ours. The stammering case study presented here uses acceptance and commitment therapy (ACT) as the evidence base [24]. The speech intervention was based on the hierarchy of production stages [27]. It is not clear what the theoretical underpinning is for the social communication group provided in the mental health inpatient unit; however, there is some evidence for the benefits of social skills training with this client group [30]. Finally, a set package of intervention was provided by the LSA to support the memory and comprehension skills in the final intervention case study; weak evidence exists on the efficacy of indirect SLT provision. McCartney et al. [31] found that intervention provided by others using a set package was not as effective as direct SLT, but part of the difference may have been due to the amount of provision offered. The paper suggests over 20 h of provision would be required to observe significant improvements, whereas less than two hours was offered in the case study presented.

There are two previously published papers [17,32] presenting a series of case studies on SLT delivery with young people in custodial settings. There are a number of differences and similarities between the current study and these papers. All present case studies of SLT delivered to young people detained in a custodial setting with complex backgrounds. However, there are some significant differences: the earlier papers come from Australia rather than England (with countries differing in their legal systems). Perhaps the greatest difference is that the intervention in the earlier studies was provided by one therapist who attended the prison specifically for research purposes, whereas the current study presents pragmatic data collected from clinicians carrying out their everyday role. Whilst this may provide a more naturalistic view of service provision, there were difficulties encountered gathering the requested data from busy clinicians. Case study templates were developed from Snow and Woodward [17] to allow for comparison; however, clinicians did not collect data for all fields and there is very limited information regarding outcome measures and service user feedback. Data was requested on outcome measures and service user feedback; however, the researcher did state this was requested ‘where available’. The information provided suggests that services do not routinely collect outcome measures and service user feedback. Outcome measure data is essential to understand what works [33].

As previous case series were delivered as research, exclusion criteria were applied to support ‘completion’ of intervention and homogeneity across the cohort [17,32]. It would not be possible or appropriate to include exclusion criteria in the current study where case studies were conducted as part of typical service provision. This does mean some of the case studies presented have an incomplete block of intervention, and there are additional cultural and language considerations to be made when devising intervention programmes. Snow and Woodward acknowledge “future researchers will need to embrace, rather than avoid such complexity” [17].

Another significant difference between the studies is the manner of assessment. For example, in Snow and Woodward [17], each individual completed an assessment battery comprised of CELF-4 [34] core language, the Test of Language Competence (TLC) [35], the La Trobe Communication Questionnaire [36], the Kaufman Brief Intelligence Test (KBIT) [37] for non-verbal IQ, the Setting Communication Goals (SCG) tool, and a therapeutic engagement tool. In the current study, the clinicians employed assessment tools specific to the client rather than applying a set battery. Whilst this leads to issues with comparing outcomes for clients, this is a more client-centred approach and reflective of usual service delivery.

Dosage is an important consideration when delivering interventions [38]. In Snow and Woodward [17], the interventions took place over 7–16 weeks, with one–two sessions per week and sessions varying from 20 to 120 min. In the current study, four of the six cases included an intervention component with the interventions taking place over 1–13 weeks with one–two sessions per week and sessions varying from 30 to 60 min. A comparison of provision across studies is shown in Table 14; the amount of intervention both in terms of number of sessions and minutes of intervention was shorter in the current study than in the published case study series. One of the conclusions from Snow and Woodward [17] was that interventions were under dosed, yet the number of sessions and the length of the intervention were both shorter in the current study. This may indicate that the current study was chronically underdosed; however, there is debate about whether more is necessarily better and whether low-frequency intervention may actually support consolidation [39]. Therefore, we must consider in clinical practice what the optimal dosage is for each individual and how we are able to provide sufficient dosage to maximise benefits.

Whilst the number and duration of sessions was lower in the current study, the number of cancellations was reduced. Provision of healthcare within prison settings is reliant upon support from the custodial staff and the prison regime, and having existing positive working relationships appears to support sessions proceeding as planned.

## 6. Limitations

There are a number of limitations to the current study, including the small number of case studies included, missing data from the case studies, lack of qualitative feedback from service users, and the inclusion of only two of the three youth prisons with SLT services. Furthermore, the data collected is reliant upon reporting from others; data is limited in terms of the description of the intervention approaches, i.e., the theoretical underpinning or why the specific intervention was chosen; also, data about the individual prior to prison is very limited. The case study data would be enhanced by the inclusion of outcome measures for both the intervention and overall well-being, as well as by long-term follow-up.

## 7. Future Directions

In the future directions section of their paper, Snow and Woodward suggest “future researchers should seek to employ more rigorous experimental design elements” [17]. This current study does the opposite; however, a strength of this design is that it provides a profile of how actual clinical services operate. There is often conflict between ‘messy’ clinical practice and ‘clean’ research design, and future studies will have to consider how to balance these two elements. Glogowska, Campbell, Peters, Roulstone, and Enderby [40], in their evaluation of service provision, assert that to truly address the question of the effectiveness of the SLT service, it is necessary to include both outcome measures and the user’s voice. In future studies, this must be thought about more carefully to ensure this data is captured.

Beyond what works, we must also consider what is acceptable. Bryan, Freer and Furlong [41] found that some commonly used assessment tools were not acceptable for this population. Given that this population is more likely to have had previous negative experiences with healthcare, and that significant power imbalances exist within prison settings, it is essential that we listen to their voice and understand their needs.

Future research should focus on beginning to develop the intervention evidence base for this client group, with careful consideration of their clinical needs, past experiences, the specific context, and acceptability. Future studies should include qualitative approaches centring on the young people and SLTs to further understand services. A survey to gather data from an international perspective could be helpful to provide a broader picture. These could then inform an experimental cohort study looking at if and how SLT interventions are effective.

## 8. Clinical Implications

Whilst very limited evidence exists regarding the efficacy of intervention in this specific client group, therapists drew on the wider evidence base to develop intervention programmes for the clients. There is not currently a specific evidence base that underpins intervention with this client group; therefore, SLTs have to apply evidence-based principles from the best available sources. SLTs working with this client group should be aware of and apply the evidence base for intervention with adolescents [42] and work with those with mental health/behavioural needs [43,44,45] where appropriate. SLTs working in this area would benefit from more evidence on interventions with adolescents and young adults and those with additional needs (e.g., behavioural and/or mental health) in the absence of evidence for this specific client group.

## 9. Conclusions

Prevalence studies repeatedly find that speech, language and communication needs are hugely overrepresented amongst children and young people in contact with the criminal justice system [1]. The criminal justice system is heavily reliant upon verbal communication skills [46], supporting the need for speech and language therapy services to be provided. This study demonstrates that speech and language therapy services can be delivered within custodial youth justice settings, with individuals making positive changes in relation to their intervention goals and more broadly. These findings support the inclusion of speech and language therapy as part of the core prison healthcare regime.

## Figures and Tables

**Table 1:** Case study overview.

	Age	Ref Source	Type of SLCN	Intervention	Mode	No of Sessions	Total Time (min)
Freddie	18;06	CHAT	Stammer	Ax + Therapy	Individual	2 + 7	510
Sam	21;05		Speech	Therapy	Individual	10	430
Brett	20;11	Primary Care	ASD	Ax	Individual	6	260
Salim	21;00	NA	Social Communication	Therapy	Group	11	630
Xavier	17;10	CAMHS	Language	Ax	Individual	1	25
Trent	18;01	CAMHS	Language	Ax + Therapy	Individual	1 + 4	155

Note: Ref source = referral source; SLCN = speech, language and communication needs; CHAT = comprehensive health assessment tool; Ax = assessment; ASD = autism spectrum disorder; NA = not applicable; CAMHS = child and adolescent mental health services.

**Table 2:** Case study template overview.

Background Information
Referral information
Assessment overview
Intervention overview
Detailed session description(s)

**Table 3:** Demographic data.

	SEN	Exclusion	MH	LAC	BAME	Violent
Freddie	Yes	Yes	Yes	NR	Yes	Yes
Sam	NR	NR	NR	NR	Yes	NR
Brett	Yes	Yes	Yes	No	No	Yes
Salim	NR	NR	Yes	NR	Yes	Yes
Xavier	No	NR	No	NR	Yes	No
Trent	NR	Yes	Yes	Yes	NR	Yes
Total	33%	50%	67%	17%	67%	67%
Total (excluding nr)	67%	100%	75%	50%	80%	80%

Note: SEN = special education needs; Exclusion = school exclusion; MH = mental health; LAC = looked after child; BAME = Black, Asian and minority ethnic groups; NR = not recorded.

**Table 4:** Freddie intervention overview.

	Sessions Offered	Sessions Completed	Duration
Assessment	2	2	90 min
Therapy	10	7	420 min
Overall	12	9	510 min

**Table 5:** Freddie therapy overview.

	Description		
Approach	Psychoeducation, diaphragmatic breathing, easy onset, soft contacts		
Aims	Improved confidence in communicating with others Increased fluency	Outcome	Achieved Partially Achieved
Method	1:1 with SLT		

**Table 6:** Sam intervention overview.

	Sessions Offered	Sessions Completed	Duration
Assessment	NA	NA	
Therapy	13	10	430 min
Overall	13	10	430 min

Note: NA; Information not available.

**Table 7:** Sam therapy overview.

	Description		
Approach	Articulation therapy, teaching memory strategies		
Aims	Production of /s/ and /z/ in phrases, sentences, and finally general conversation Learn strategies to assist with auditory and visual memory.	Outcomes	Achieved Achieved
Method	1:1 with SLT		

**Table 8:** Brett intervention overview.

	Sessions Offered	Sessions Completed	Duration
Assessment	6	6	260 min
Therapy	NA	NA	
Overall	6	6	260 min

Note: NA; Information not available.

**Table 9:** Salim intervention overview.

	Sessions Offered	Sessions Completed	Duration
Assessment	1	1	30 min (approx.)
Therapy	12 (approx.)	10 (approx.)	600 min (approx.)
Overall	13	11	630 min

**Table 10:** Salim therapy overview.

	Description		
Approach	Weekly social communication group tailored to individuals’ functional goals		
Aims	To encourage and support Salim in practising and developing good communication skills Having communication skills required to complete driving theory test	Outcomes	Partially achieved Partially achieved
Method	Group facilitated by student SLT and prison officer/mental health nurse		

**Table 11:** Xavier intervention overview.

	Sessions Offered	Sessions Completed	Duration
Assessment	1	1	25 min
Therapy	Unknown	Unknown	
Overall	1+	1+	25 min+

**Table 12:** Trent intervention overview.

	Sessions Offered	Sessions Completed	Duration
Assessment	1	1	15 min
Therapy	4	Unknown	140 min (approx.)
Overall	5	Unknown	155 min (approx.)

**Table 13:** Trent therapy overview.

	Description		
Approach	Inferential comprehension tasks		
Aims	To work on increasing vocabulary To develop memory/understanding	Outcomes	Unknown Unknown
Method	Indirect via 1:1 with Learning Support Assistant		

**Table 14:** Case study comparison.

	Snow and Woodward [17]	Swain, Eadie and Snow [31]	Current Study
BAME/ATSI	67%	0%	75%
No. of sessions	18.5	16.75	8.75
No. of sessions cancelled	24.5%	33.92%	13%
No. of minutes of intervention	797	NR	345

Note: BAME = Black, Asian and minority ethnic groups; ATSI = Aboriginal and Torres Strait Islanders; NR = not reported.

## Data Availability

The data presented in this study are openly available in [White Rose etheses, https://etheses.whiterose.ac.uk/id/eprint/24922/1/Final%20thesis%20COMPLETE.pdf] (accessed on 25 August 2026).

## Supplementary Materials

The following supporting information can be downloaded at: https://www.mdpi.com/article/10.3390/healthcare14172882/s1, File S1: Phase 3: Case Study Template. References [17,47] are cited in the Supplementary Materials.
