# Supplementary material for: Profiling Speech and Language Therapy Delivery in Custodial Youth Justice Settings in England: A Case Study Series
Source: Healthcare (Basel). 2026 Sep 7;14(17):2882. doi: 10.3390/healthcare14172882 (PMC13566015; doi:10.3390/healthcare14172882)
Supplement: Supplementary file 1 [file healthcare-14-02882-s001.zip › healthcare-4370206-supplementary.pdf]

Phase 3: Case Study Template

Overview

Age: (month, years)

Schooling: Exam results, Expelled (+ age), Years of schooling, SEN, Statement/EHC plan, ESL

Mental Health: Diagnoses, ADHD, ASD, Substance Misuse, Previous contact with services

Family & Society: LAC, SES, Child Protection, BME, Similar Peers

Offending: Index Offence Category, Length of stay, Violent v Non Violent

Physical Health: TBI

Referral

Referral source:

Reason for referral:

Assessment

Assessment tools:

Assessment summary:

Primary areas of need:

Time taken for assessment: (no of sessions and minutes)

Therapy

Treatment aims:

Intervention approach:

Sessions: (no of sessions and minutes)

Sessions cancelled: (and reason)

Weeks in therapy:

Therapy provider: (include whether 1:1 or prison officer/assistant sitting in)

Session description

Took place/Cancelled (if cancelled give reason)

Where:

Room set up: (who present, how seated, furniture)

Target:

Achieved/Not achieved

Materials:

General presentation:

Progress:

References

Marks, I., & Stokes, S. F. (2010). Narrative-based intervention for word-finding difficulties: A case study. *45*(5), 586-599.

Snow, P. C., & Woodward, M. N. (2016). Intervening to address communication difficulties in incarcerated youth: A Phase 1 clinical trial. *International Journal of Speech-Language Pathology*, 1-15. doi:10.1080/17549507.2016.1216600
